# Cortico-Cerebellar neurodynamics during social interaction in Autism Spectrum Disorders

**DOI:** 10.1016/j.nicl.2023.103465

**Published:** 2023-06-28

**Authors:** Fleur Gaudfernau, Aline Lefebvre, Denis-Alexander Engemann, Amandine Pedoux, Anna Bánki, Florence Baillin, Benjamin Landman, Anna Maruani, Frederique Amsellem, Thomas Bourgeron, Richard Delorme, Guillaume Dumas

**Affiliations:** a Human Genetics and Cognitive Functions, Institut Pasteur, UMR 3571 CNRS, University Paris Diderot, Paris, France; b Inria, HeKA, PariSantéCampus, Paris, France; c Inserm, Centre de Recherche des Cordeliers, Sorbonne Université, Université de Paris Cité, Paris, France; d Department of Child and Adolescent Psychiatry, Robert Debré Hospital, APHP, Paris University, Paris, France; e Roche Pharma Research and Early Development, Neuroscience and Rare Diseases, Roche Innovation Center Basel, F. Hoffmann–La Roche Ltd., Basel, Switzerland; f Université Paris-Saclay, Inria, CEA, Palaiseau, France; g Research Unit Developmental Psychology, Department of Developmental and Educational Psychology, Faculty of Psychology, University of Vienna, Vienna, Austria; h Department of Psychiatry, Faculty of Medicine, Université de Montréal, Montréal, QC, Canada; i Precision Psychiatry and Social Physiology laboratory, CHU Sainte-Justine Research Centre, Université de Montréal, Montréal, QC, Canada

**Keywords:** Neurodevelopment, Social Cognition, Theta Oscillations, Cerebellum, Digital Psychiatry, Autism, Human-machine interaction (HMI)

## Abstract

**Background:**

Exploring neural network dynamics during social interaction could help to identify biomarkers of Autism Spectrum Disorders (ASD). A cerebellar involvement in autism has long been suspected and recent methodological advances now enable studying cerebellar functioning in a naturalistic setting. Here, we investigated the electrophysiological activity of the cerebro-cerebellar network during real-time social interaction in ASD. We focused our analysis on theta oscillations (3–8 Hz), which have been associated with large-scale coordination of distant brain areas and might contribute to interoception, motor control, and social event anticipation, all skills known to be altered in ASD.

**Methods:**

We combined the Human Dynamic Clamp, a paradigm for studying realistic social interactions using a virtual avatar, with high-density electroencephalography (HD-EEG). Using source reconstruction, we investigated power in the cortex and the cerebellum, along with coherence between the cerebellum and three cerebral-cortical areas, and compared our findings in a sample of participants with ASD (n = 107) and with typical development (TD) (n = 33). We developed an open-source pipeline to analyse neural dynamics at the source level from HD-EEG data.

**Results:**

Individuals with ASD showed a significant increase in theta band power over the cerebellum and the frontal and temporal cortices during social interaction compared to resting state, along with significant coherence increases between the cerebellum and the sensorimotor, frontal and parietal cortices. However, a phase-based connectivity measure did not support a strict activity increase in the cortico-cerebellar functional network. We did not find any significant differences between the ASD and the TD group.

**Conclusions:**

This exploratory study uncovered increases in the theta band activity of participants with ASD during social interaction, pointing at the presence of neural interactions between the cerebellum and cerebral networks associated with social cognition. It also emphasizes the need for complementary functional connectivity measures to capture network-level alterations. Future work will focus on optimizing artifact correction to include more participants with TD and increase the statistical power of group-level contrasts.

## Graphical abstract

**Figure:**
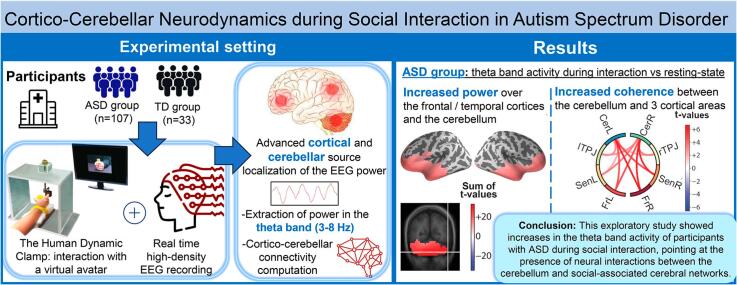

## 1 Introduction

Autism Spectrum Disorders (ASD) are neurodevelopmental disorders characterized by impaired social interactions and repetitive, stereotyped behaviors (Diagnostic and Manual, 2013). As the diagnosis of ASD primarily relies on clinical assessment, identifying robust biomarkers, specifically of neural dynamics during social interaction, could help achieve an earlier and more consistent diagnosis (Lord et al., 2000). Magnetoencephalography (MEG) and electroencephalography (EEG) are optimal for functional analysis (Khan et al., 2015, Mostofsky and Ewen, 2011), thanks to their millisecond time resolution and ability to explore large frequency bands - such as 0.5–60 Hz for EEG vs. 0.01–0.1 Hz in functional magnetic resonance imaging (fMRI). Most quantitative EEG studies in ASD have focused on brain activity during resting state, suggesting oscillatory anomalies that draw a U-shaped profile with greater relative power in low-frequency and high-frequency bands (Wang et al., 2013). Although some studies have started investigating brain signals during action observation (Oberman et al., 2005, Dumas et al., 2014b), no EEG study has been conducted during real-time social interaction in ASD (Lord et al., 2020).

We recently developed a novel paradigm, the Human Dynamic Clamp (HDC), for studying real-time social interactions based on interpersonal coordination between a human and a Virtual Partner (VP) (Dumas et al., 2014a). People coordinated hand movements with the observed movements of a virtual hand, the parameters of which depended on the real-time input from the participant’s own movements. Neural dynamics could be recorded in parallel with high-density (HD)-EEG, thus providing insights into the dynamics of real-time social interactions in a controlled experimental setting, since the social interaction variability depended only on the participant (Dumas and Fairhurst, 2021). The HDC task in participants with typical development (TD) revealed local recruitment in the theta band activity in the motor areas during movement execution and in the right parietal areas during coordination with the virtual hand, as well as distributed connectivity across the antero-posterior network during intention attribution to the VP (Dumas et al., 2020). Recent empirical work with the HDC in ASD provided preliminary evidence of a relationship between sensory-motor and socio-cognitive impairments (Baillin et al., 2020).

Recently, the link between ASD and the cerebellum, which has a critical role in social interactions (Van Overwalle et al., 2019), has attracted growing interest. The cerebellum contributes to the understanding and imitation of others' actions by observing their body (Ramsey et al., 2021) and mentalizing, *i.e.* inferring another person’s state of mind (Van Overwalle et al., 2014). It is also involved in the neural encoding of rhythmic processes (Nozaradan et al., 2017). While cerebellar atypicality has been reported in individuals with ASD (Baron-Cohen et al., 2008), a recent study (Laidi et al., 2022) conducted on a larger cohort found no difference in cerebellar anatomy between individuals with ASD and individuals with TD. Investigating cerebellar differences at the functional level might be a more promising research avenue (Dong et al., 2022). Numerous neuroimaging studies have described abnormal cerebellar activation in participants with ASD during motor tasks and visual processing tasks (Philip et al., 2012). Despite its limited temporal resolution, most of these studies have primarily investigated cerebellar activity using fMRI. To our knowledge, EEG-based studies conducted with ASD participants to date focused on cortical signals and did not include the cerebellum (Wang et al., 2013). However, studies conducted in adults with TD demonstrated that both EEG and MEG can detect signals from the cerebellum (Samuelsson et al., 2020).

In this study, we investigated the EEG of the cerebellum during an interactive social interaction task in ASD using the HDC paradigm. We specifically focused our analysis on the theta frequency band (3–8 Hz) (Aykan et al., 2022), which has been detected in the cerebellum (D'Angelo et al., 2009), is associated with large-scale coordination of distant brain areas (Moreau et al., 2018), and might contribute to interoception, motor control, and social event anticipation, all skills critically affected in ASD (Lord et al., 2020). As previous EEG and MEG studies consistently reported atypical cortical theta power in children with ASD during resting state (Coben et al., 2008, Cornew et al., 2012), we expected abnormal theta activity both at the cortical and cerebellar level in individuals with ASD.

Beyond investigating cerebellar power spectra, we further aimed to describe the connectivity patterns between the cerebellum and the sensorimotor cortex but also the prefrontal cortex (PFC) and the temporoparietal junction (TPJ). During tasks in which adults with TD had to observe repetitive hand movements, MEG analysis uncovered high connectivity between the cerebellum and the primary motor cortex in low-frequency EEG bands (<5 Hz) (Bourguignon et al., 2013). Additional evidence suggested that the cerebellum was connected to the prefrontal cortex (PFC) and involved in motor planning; as well as to the right TPJ (rTPJ), participating in forming inferences on others’ intentions (Van Overwalle and Mariën, 2016). Thus, we expected atypical connectivity in the theta frequency band between the cerebellar and cerebral cortices during social interaction. To address both of our hypotheses, we used state-of-the-art source reconstruction methods to locate surface cortical and deep cerebellar sources of the recorded EEG activity.

## 2 Materials and methods

### 2.1 Participants

The experiment was conducted on a sample of 217 individuals, including 161 with ASD and 56 with TD.

Participants with ASD were recruited at the Child and Adolescent Psychiatry Department of the Robert Debré University Hospital, Paris (France), and were included after a systematic clinical and medical examination, including negative blood test results for Fragile X syndrome. The diagnosis of ASD was performed by certified clinical psychiatrists. It was based on the Diagnostic and Statistical Manual of Mental Disorders (DSM-5) criteria and outcomes from the Autism Diagnostic Observation Schedule-Second Edition (ADOS-II) (Lord et al., 2012), the Autism Diagnostic Interview-Revised (ADI-R) (Lord et al., 1994), and the Social Responsiveness Scale–Second Edition (SRS-2) (Constantino and Volkmar, 2013) for the dimensionality of social skill impairments. Participants carrying a large deletion over 2 million base pairs (Mb) detected by the Illumina 700 SNPs genotyping array and those with a medical history of epileptic seizures were excluded. The final sample for analysis included only individuals without an intellectual disability estimated with the Wechsler Intelligence Scale (Wechsler, 2014) adapted to the participant's age. Participants with TD were recruited from the general population, and also reported no personal or familial history of ASD and/or epileptic seizures.

This study was carried out in accordance with the recommendations of the local ethics committee of the Robert Debré Hospital (study approval no. 08–029). All participants and caregivers of child participants gave written informed consent in accordance with the Declaration of Helsinki.

### 2.2 Experimental procedure

**Fig. 1:**
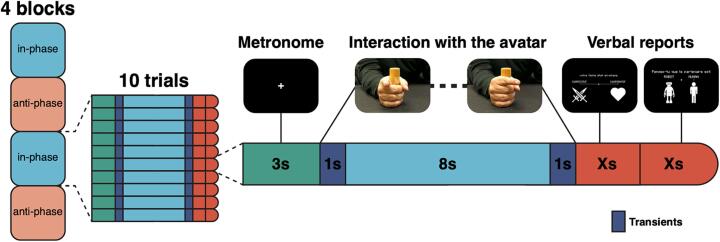
Experimental design. Structure of the protocol with four blocks alternating ‘in-phase/anti-phase’ (the order of blocks was counterbalanced across participants). Each block was divided into ten trials (left). Each trial started with a ‘warm-up’ phase in which the participant had to synchronize finger movements with the sound of a metronome for 3 seconds. Then, the participant interacted with the avatar according to the instruction (*i.e.* here, ‘in-phase’). The 1-second transient periods prevented integrating non-stationary dynamics when switching between the metronome and interaction periods. We present screenshots of what participants could see (top).

The experimental design consisted of a resting state (RS) acquisition period and a social interaction task based on the HDC paradigm that all participants completed, while their neural dynamics were recorded with HD-EEG. The RS served as a control condition for comparison with the HDC experimental condition. During the RS protocol, the participant was requested to sit still and keep his/her eyes open, then closed for 30–30 s (conditions ‘eyes-open’ and ‘eyes-closed’). This series of two conditions was also repeated three times. The HDC protocol used a human–machine interface as initially described in Dumas et al., 2014a (Fig. 1). As a summary, the HDC relies on a mathematical model integrating the position and speed of the participant’s index finger during movement to simulate the behavior of a VP in real-time. At the beginning of each trial, an instruction (with respect to language comprehension abilities) was given to the participant by the experimenter to synchronize index finger movement ‘in-phase’, *i.e.* matching her/his movements to those of the VP, or in ‘anti-phase’, *i.e.* to synchronize her/his movements with a half-period offset between the phase of the VP and the participant. The protocol consisted of 40 trials of about 20 second each, divided into four blocks. The instructions to the participant stayed the same within each block and alternated across blocks. The instruction for the first block was randomly assigned at the beginning of the experiment to ensure randomized order of blocks across participants.

### 2.3 HD-EEG acquisition

HD-EEG was recorded by a 128-channel Geodesic EEG System 400 (GES 400) with NetStation EEG software and a 128-electrode HydroCel Geodesic Sensor Net (EGI, Inc., Eugene, OR, USA). The sensor net was connected to a Net Amps 400 amplifier (EGI, Inc., Eugene, OR, USA), which was set to ‘255 encoding mode’ for recording external Digital Input (DIN) events (triggers). EEG data were amplified, low-pass filtered at 500 Hz, sampled at a frequency of 1000 Hz, and digitized. For the RS protocol, an event list was created in the EEG recording software with a label for each condition, which was manually selected before each task by the experimenter. For the HDC protocol, DIN events were recorded automatically. After the HD-EEG recording, the EEG electrode locations were digitized using a stereo-camera tracking digitizer (GeoScan Sensor Digitization Device, 397 Philips Neuro Inc., Eugene, OR).

### 2.4 Data preprocessing

HD-EEG data were preprocessed offline with the open-source software package MNE-Python (Gramfort et al., 2013) to attenuate noise and artifacts from environmental and biological sources. Preprocessing started with defining events based on markers (triggers). Next, data were band-pass filtered to restrict the signal to the frequency range of interest (from delta [1–4 Hz] to gamma [30–45 Hz] waves), between 0.5 and 48 Hz. For that, MNE-Python default parameters were used, *i.e.* a fast Fourier transform-based Finite Impulse Response filter with a Hamming window. To avoid signal contamination from the reference or any malfunctioning channel, the signal was averaged and referenced using the Signal-Space Projection method. Then, raw data were segmented into 1-second-long epochs from both protocols (HDC, RS), epochs were decimated by a factor of 4 and the automated artifact rejection algorithm Autoreject was applied to reject or repair noisy epochs, based on channels’ trial-wise peak-to-peak amplitude (Jas et al., 2017). Channel-wise thresholds were computed with Bayesian optimization; other parameters were set as default. When the peak-to-peak signal amplitude exceeded a data-specific threshold, epochs with noisy signals were rejected. When possible, data from these epochs were interpolated and replaced within the whole length of the signal. The algorithm was first trained on epochs from both protocols (HDC, RS). Afterwards, for each protocol, relevant epochs were extracted and cleaned by the previously trained algorithm according to the above parameters.

To further assess the quality of the EEG data, we checked for the accuracy of our data to replicate the most robust findings from the literature, both at source and sensor levels. Since these preliminary analyses impacted subsequent methodological choices, and in order to favor a linear narrative, the preliminary results will be presented in the Methods section.

### 2.5 Sensor analysis

At sensor level, we expected the Power Spectral Density (PSD) in the alpha band (8–13 Hz) to increase in the ‘eyes-closed’ condition. In our study, we similarly observed an increased activity in the alpha band over the whole scalp, both in participants with ASD and TD; this increase of alpha power was significantly lower in the prefrontal cortex of children with ASD compared to those with TD (Sup. Fig. S1). We also expected to replicate a mu suppression (*i.e.* reduction of the oscillations in the alpha frequency range over the sensorimotor cortex in a social context) in the upper alpha band (10–13 Hz) during the HDC task (Kirschfeld, 2005, Dumas et al., 2014c). Mu activity was indeed suppressed over the scalp in both groups with TD and ASD during social interaction (Sup. Fig. S2). No statistically significant difference in mu activity was observed between groups.

### 2.6 Source reconstruction

We reconstructed the location and spectral content of sources in the brain using MNE-Python (Gramfort et al., 2013). Coregistration was performed to bring into alignment the digitized sensor locations and the MNE-Python MRI template brain. Sources were reconstructed on this template brain using a mixed source space. The noise-covariance matrix was then calculated from the RS ‘eyes-open’ condition with shrinkage regularization. Proper estimation of the covariance matrix is one of the most critical factors in the accuracy of the reconstruction (Engemann et al., 2015). The inverse solution was computed using the Exact Low Resolution Brain Electromagnetic Tomography (eLORETA) algorithm (Pascual-Marqui et al., 2011).

To assess the quality of source estimation by the eLORETA algorithm, analysis of alpha power during the ‘eyes-closed’ condition was replicated over the cortex after source reconstruction performed by both eLORETA (Pascual-Marqui, 2007) and Minimum Norm Estimate (MNE) algorithms (Sup. Fig. S1 and Sup. Fig. S3, respectively). In addition, PSD comparison in the alpha band between HDC and RS was performed over the cortex. This allowed us to check if mu suppression was also replicated at the source level, hence providing another way of validating the results of source reconstruction. This mu suppression analysis was performed on signals reconstructed with a noise covariance matrix low-pass filtered at 8 Hz in order to include only low frequencies in the noise model and avoid considering intrinsic alpha brain activity as noise. After source reconstruction with eLORETA, we observed that the alpha power increased significantly over the occipital lobe in both groups with ASD and TD in the ‘eyes-closed’ condition (Sup. Fig. S1). No cluster was specifically associated with ASD. Similar results were observed after source reconstruction using the MNE algorithm (Sup. Fig. S3). We then compared EEG signals during the social interaction (HDC) task vs. the RS ‘eyes-open’ condition. We observed a significant alpha power decrease all over the cerebral cortex in individuals with ASD and TD (Sup. Fig. S2). We however reported no clusters specifically associated with ASD.

### 2.7 Functional connectivity

Following source reconstruction, source time courses were averaged inside regions of interest (ROIs) following the Desikan-Killiany atlas (Desikan et al., 2006). Functional connectivity was calculated using the coherence metric (Maris et al., 2007) between pairs of ROIs (with 125 FFT length, and 62 overlap between segments):

$$\mathrm{coh}S_{\mathrm{xy}}\left(f\right)=\frac{|S_{\mathrm{xy}}\left(f\right)|}{\sqrt{{(S}_{\mathrm{xx}}\left(f\right)S_{\mathrm{yy}}\left(f\right))}}$$

With *x* and *y* the indices of two ROIs, *f* the frequency of interest, $S_{\mathrm{xy}}$ the cross-spectral density between *x* and *y*, $S_{\mathrm{xx}}$ and $S_{\mathrm{yy}}$ the power spectrums of *x* and *y* (respectively).

To increase the robustness of our analysis and mitigate the effects of spatial leakage, we performed symmetric orthogonalisation with MNE-Python (Maris et al., 2007) and recomputed coherence values from the orthogonalized source time courses.

We also computed a phase-based connectivity metric, the weighted Phase Lag Index (wPLI) (Vinck et al., 2011):

$$wPLI(S_{xy}\left(f\right))=\frac{Im(S_{xy}\left(f\right))}{\left|Im(S_{xy}\left(f\right))\right|}$$

With *Im* the Imaginary part of a complex number.

For each participant, PSD and connectivity values were averaged over the frequency band of interest and Z-scored over epochs to represent the statistical distance between conditions and account for intra-subject variability. The Z-connectivity was defined as:

$$Z_{\mathrm{xy}}\left(f\right)=\frac{\mu_{\mathrm{xy}}^{A}\left(f\right)-\mu_{\mathrm{xy}}^{B}\left(f\right)}{\sigma_{\mathrm{xy}}^{B}\left(f\right)}$$

with $\mu_{\mathrm{xy}}^{A}$ and $\mu_{\mathrm{xy}}^{B}$ the average connectivity between *x* and *y* over epochs in A (HDC) and B (RS ‘eyes-open’) conditions, and $\sigma_{\mathrm{xy}}^{B}$ the standard deviation of the connectivity in condition B over epochs.

### 2.8 Statistical analysis

Statistical analyses were performed with MNE-Python. The power spectra of the cortex and the cerebellum were compared between groups and between conditions both at sensor and source level. Cluster-based permutation tests were used to correct for multiple comparisons over the entire brain surface (Maris and Oostenveld, 2007, Pantazis et al., 2005). Cluster permutation tests combined spatially neighboring voxels into clusters and computed a cluster-level statistic. Permutations (n = 2000) randomly exchanged the labels of participants to estimate the distribution of the maximum cluster statistics under the null hypothesis of no difference between groups or conditions. A cluster was considered statistically significant if its statistic was greater than 95% of the maximum cluster statistics that would be expected by chance. We also used threshold-based free cluster enhancement (TFCE) correction (Smith and Nichols, 2009), which enhanced areas of the signal belonging to cluster-like regions. Effect sizes (Z-scores for within-group tests and Cohen’s d for between-group tests) were also computed for each analysis.

Functional connectivity between the cerebellum and cortical areas during social interaction was analysed at the source level. Functional connectivity in the theta band between the cerebellum and three cortical areas (sensorimotor cortex, PFC and TPJ) during interaction with the HDC was compared between groups using Student t-tests with false discovery rate (FDR) correction for multiple comparisons. In power and connectivity analyses, effect sizes were computed for intra- and inter-group comparisons to reduce the unequal sample size bias. Test statistics were displayed for the intra-group comparisons with statistically significant results.

Based on the sample size of the two groups (N_ASD_ = 107; N_TD_ = 33), we calculated the statistical power of the difference between two independent means (two-tails) using the software G*Power (Faul et al., 2007). With a significance threshold of alpha = 0.05 and an estimated effect size from the literature of d = 0.5 (Loth et al., 2021), we obtained a statistical power of 1-beta = 0.7.

## 3 Results

### 3.1 EEG power analysis

**Fig. 2:**
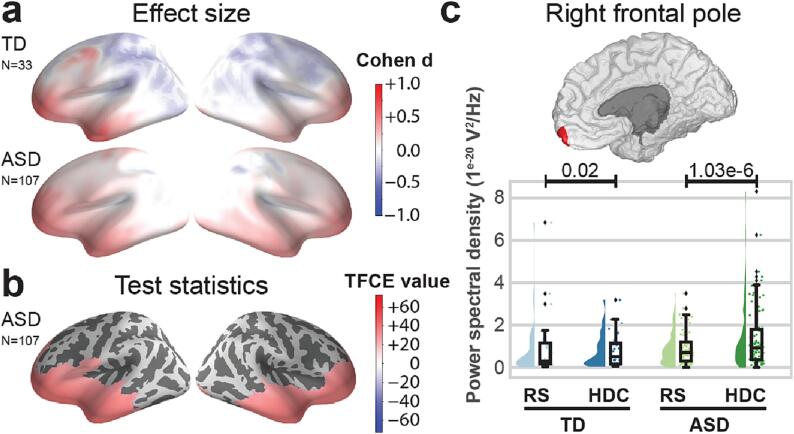
During the HDC task, cerebral cortex effect sizes and statistical maps are compared to the ones obtained in the RS ‘eyes-open’ condition for the theta band (3–8 Hz). Panels (a) and (b) show brain plots displaying the activity of the left and right hemispheres. Panel (a) shows effect sizes for the TD group (upper) and the ASD group (lower). Positive effect sizes indicate theta enhancement during the HDC. Panel (b) shows TFCE values in the group with ASD thresholded at p < 0.05 corrected. Positive values indicate greater theta enhancement during the HDC. On panel (c), half violin plots display PSD values in the right frontal pole in the TD (blue) and ASD (green) groups during RS (light) and HDC (dark) (Desikan et al., 2006). For each group and condition, a density plot and a boxplot are displayed on the left and right (respectively). P-values indicate the results of intra-group comparisons (thresholded at p < 0.05, non-corrected).

**Fig. 3:**
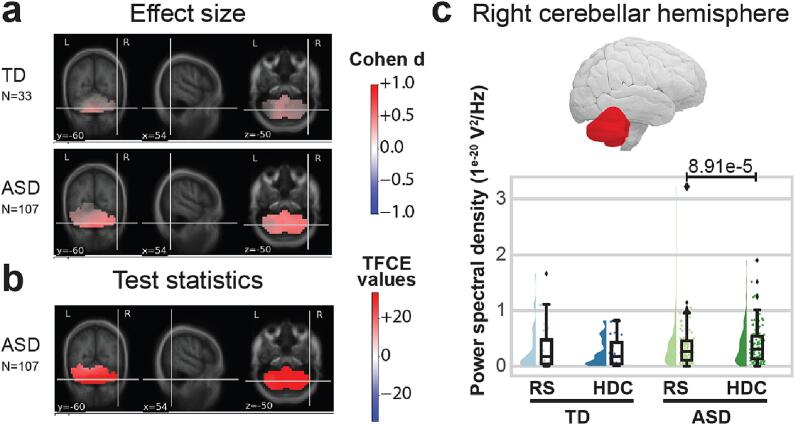
During the HDC task, the cerebellar cortical effect sizes and statistical maps are compared to those obtained in the RS ‘eyes-open’ condition for the theta band (3–8 Hz). On panels (a) and (b), the cerebellar activity is displayed on the coronal (left), sagittal (middle), and transverse (right) sections. Panel (a) shows effect sizes for the TD group (upper) and the ASD group (lower). Positive effect sizes indicate theta enhancement during the HDC in the TD and ASD groups (respectively). Panel (b) shows TFCE values in the group with ASD thresholded at p < 0.05, corrected. On panel (c), half violin plots display PSD values in the right cerebellar hemisphere in the TD (blue) and ASD (green) groups during RS (light) and HDC (dark). For each group and condition, a density plot and a boxplot are displayed on the left and right (respectively). P-values indicate the results of intra-group comparisons (thresholded at p < 0.05, non-corrected).

To ensure the robustness of the source reconstruction, Cohen’s d effect size was calculated for each dipole location and each participant between the HDC and RS conditions in the theta band. Participants with more than 5% sources with a Cohen’s d value above 10 were considered as outliers. We finally ran the analysis on 140 participants, *i.e.* 107 with ASD and 33 with TD. Demographic characteristics of the participants are available in Table 1. In the analysis of theta power (Fig. 2), we observed a significant increase in theta power in the temporal and frontal areas in the ASD group (TFCE value at peak = 72.68; p-value at peak = 0.01) during social interaction. We observed no statistically significant clusters in the TD group, nor in the ASD vs. TD groups contrast. Finally, we observed a significant increase in theta power in both cerebellar hemispheres in participants with ASD (TFCE value at peak = 33.77; p-value at peak = 5e-4) (Fig. 3). We found no statistically significant clusters associated with TD, nor with the ASD vs. TD groups contrast. The analyses of theta power over the cortex and the cerebellum were replicated with a p-threshold of 0.01, which yielded comparable results.

**Table 1:** Demographic and clinical characteristics of participants included in the study. Data are mean values ± standard deviation and (minimum–maximum values) for continuous variables.

	individuals with ASD (n = 107)	individuals with TD (n = 33)	Statistical test; p-value
Sex (male/female)	90/16	18/13	χ^2^ = 16.11; p = 6.71e-5
Age at inclusion (years)	13.16 ± 8.11 (3–45)	19.22 ± 9.68 (6–53)	t = -3.55; p = 5.24e-4
Wechsler Scale - standardized total score	102.34 ± 17.23 (71–143)	109.91 ± 13.52 (83–139)	t = -2.25; p = 2.60e-2
SRS-2 - Total t-score	70.49 ± 14.16 (39–91)	45.71 ± 8.07 (30–69)	t = 9.21; p = 8.14e-16
Right-handedness (right/left)	85/11	26/4	χ^2^ = 0.002; p = 0.75

ASD = Autism Spectrum Disorders; n = sample size; SRS-2 = Social Responsiveness Scale − 2nd Edition; TD = Typical Development. The SRS-2 data were available for only n = 81 participants with ASD and n = 18 participants with TD.

### 3.2 Connectivity analysis

**Fig. 4:**
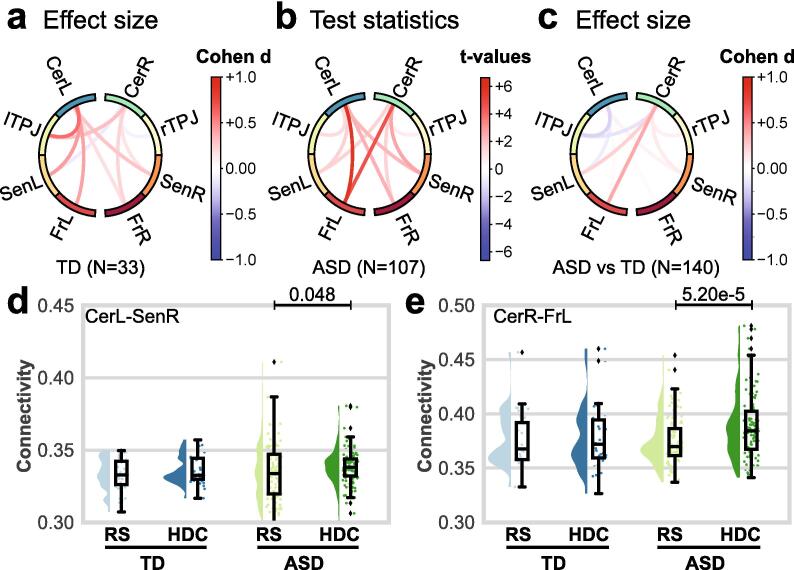
During the HDC task, the coherence effect sizes and statistical plots were compared to those obtained in the RS ‘eyes-open’ condition for the theta band (3–8 Hz). Panels (a), (b) and (c) display a circle plot with connections between different brain areas, from top to bottom: cerebellar cortices (CerL/CerR), temporo-parietal junctions (lTPJ/rTPJ), sensorimotor cortices (SenL/SenR), and frontal cortices (FrL/FrR). Panels (a) and (c) show effect sizes for the TD group and ASD vs. TD groups contrasts (respectively). Panel (b) shows connectivity T-values for the group with ASD thresholded at p < 0.05, FDR corrected. On panel (d), half violin plots display coherence values between the left cerebellar cortex and the right sensorimotor cortex in the TD (blue) and ASD (green) groups during RS (light) and HDC (dark). On panel (e), half violin plots display coherence values between the right cerebellar cortex and the left frontal cortex in the TD and ASD groups during RS and HDC. For each group, a density plot and a boxplot are displayed on the left and right (respectively). P-values indicate the results of intra-group comparisons (thresholded at p < 0.05, non-corrected).

We explored the connectivity between the cerebellar cortices and three main brain areas: the PFC and the TPJ, which both showed an increased power activity in the theta band during the social interaction task, and the sensorimotor cortex. We observed statistically significant increases in coherence between the two cerebellar hemispheres and these cortical brain areas in individuals with ASD during social interaction (Fig. 4). There were no statistically significant changes in coherence in participants with TD or differences between groups. After correction for spatial leakage, we observed statistically significant coherence increases between the left frontal cortex and the cerebellar cortices in individuals with ASD (Sup. Fig. S4).

We also computed the wPLI, a phase-based connectivity measure that is less impacted by power changes. In contrast to the analysis of coherence, we observed statistically significant cortico-cerebellar wPLI decreases in participants with ASD (Sup. Fig. S5).

## 4 Discussion

In this exploratory study, we addressed the methodological challenge of probing the role of the cerebellum in ASD during a social interaction task. We exploited recent methodological innovations, *i.e.* the HDC paradigm combined with HD-EEG and cerebellar source localization, and reported an increase of theta power in the cerebellum and in the frontal and temporal cortices, associated with an increased cortico-cerebellar coherence during movement in individuals with ASD.

The first achievement of our study was to localize cerebellar sources based on HD-EEG recordings, which was considered highly challenging (Samuelsson et al., 2020). We detected increased theta band power in the right hemisphere of the cerebellar cortex, *i.e.* ipsilateral to motor movement during interaction compared to RS. These findings are consistent with those from preliminary studies with neurotypical participants in which the contralateral sensorimotor cerebral cortex and the ipsilateral cerebellar cortex synchronically oscillated at the frequency of movement (Andersen et al., 2020). As our study was conducted during a social interaction task, comparisons with previous studies in the literature are limited. The increased cerebellar theta power we observed in our study was only significant in individuals with ASD. This pattern, uncovered during a standardized social interaction task, is consistent with the role of the cerebellum as a regulator of social behaviors (Cutando et al., 2022), and with its involvement in social cognition alterations in ASD (Wang et al., 2014). We similarly observed a significant theta power increase over frontal and temporal areas in individuals with ASD, which could foster the hypothesis of a ‘social brain network’ alteration in ASD (Sperdin et al., 2018). Our results stress further the diversity of brain structures involved in ‘low-level’ social interactions, such as a motor synchronization task.

The second achievement of our study was to explore the cortico-cerebellar connectivity during a social interaction task using the HDC paradigm. We observed statistically significant increases in cortico-cerebellar coherence in participants with ASD. After correction for spatial leakage, we still observed significant interactions between the cerebellar cortices and the left prefrontal cortex. This more stringent analysis demonstrated robust evidence for the co-activation of at least two networks during social interaction in participants with ASD. Our results reinforce previous findings in adults with neurotypical development undergoing the same HDC protocol (Dumas et al., 2014a) and are consistent with reports of increased coherence between the cerebellum and the sensorimotor cortices during movement (Bourguignon et al., 2013). Based on effect size comparisons, the coherence between the sensorimotor cortices and the right cerebellar cortex in participants with ASD appeared higher during social interaction than in those with TD. Future research with a larger sample of participants with TD is needed to ascertain this result, which is in line with previous resting state fMRI studies showing hyperconnectivity patterns between the cerebellum and sensorimotor areas in participants with ASD compared to controls (Khan et al., 2015, Oldehinkel et al., 2019).

Interestingly, these results do not align with those yielded by a phase-based connectivity metric, which showed statistically significant decreased cortico-cerebellar connectivity in the ASD group during social interaction. Since coherence and wPLI reflect different hypotheses and capture different types of information (*i.e.* phase and amplitude information versus non-zero lag phase information), it is not unexpected to obtain different results (Bastos and Schoffelen, 2016). In an interactive setting such as the HDC, meaningful neural interactions with no or minimal phase delay can actually exist, e.g. like when people synchronize (Dumas et al., 2010, Sadaghiani et al., 2012). Our results stress the importance of exploiting the complementary features of different functional connectivity metrics and the need to interpret them with caution (Colclough et al., 2016).

Our study should be considered in light of its limitations. There were major group differences in terms of age (mean = 13.16 in the ASD group vs. 19.22 in the TD group) and sex (with 15% and 42% females in the ASD and TD groups, respectively). Altered connectivity findings in ASD are not uniform across age and sex (Schwartz et al., 2017), thus some of the atypical neural dynamics we reported might arise from a sampling bias in our study. Age and gender were integrated as confounding factors in two linear regression models with the average theta power over the parietal cortex (respectively over the whole cerebellum) as dependent variables. We found no statistically significant effect of age and gender, suggesting limited confounding effects on brain activity. Participants in the ASD group also had relatively weak autistic traits (mean SRS-2 score of 70.49), which might have created less salient differences between the two groups. Dimensional analyses were precluded by large numbers of missing values (such as in the SRS-2 scale, see Table 1) and the study sample size. Further analyses will include dimensional clinical scales to perform finer analysis by recovering missing values and including more participants in the study. Second, we used a normalized adult brain template to perform source localization. This might have created a bias as we did not consider the variability linked to brain size changes with age, nor the neuroanatomical heterogeneity associated with ASD (Zabihi et al., 2019). Finally, we excluded from the analysis a large number of participants (n = 77), especially in the TD group, by using a stringent algorithm to detect EEG artifacts. This resulted in unbalanced sample sizes, which might account for the non-significant results of the within-group and group-level comparisons (statistical power = 0.7 for an effect size of 0.5). The EEG dataset included the pilot EEG data recorded on TD participants which contained on average lower quality recordings, e.g. with bad temporal markers, environmental artifacts or poor electrode contact and more often incomplete and unexploitable HDC protocol compared to RS. The development of new tools for artifact correction would enable us to include an increased number of data segments, limit the sampling bias and increase the statistical power of the study.

In conclusion, our results provided additional support for tracking cortico-cerebellar dynamics during real-time social interaction in a clinical pediatric setting. This exploratory study paves the way for multimodal interactive psychometrics for the fine-grained assessment of social skills and their underlying neural mechanisms in ASD (Baillin et al., 2020, Mobbs et al., 2021). Further work will need to integrate behavioral and motor data from the HDC to investigate how brain dynamics linked to sensorimotor coordination and intention attribution might be altered in ASD (Dumas et al., 2020). To identify robust biomarkers of spontaneous social interaction, we stress the importance of comparing complementary functional connectivity measures (Lefebvre et al., 2018). From a broader perspective, identifying neural biomarkers during naturalistic dynamic interactions could provide additional targets for therapies in children with ASD, from robot- and avatar-mediated interventions (Ali et al., 2019) to neuromodulation approaches (Dumas, 2022).

## CRediT authorship contribution statement

**Fleur Gaudfernau:** Conceptualization, Formal analysis, Software, Writing – original draft, Visualization. **Aline Lefebvre:** Investigation, Writing – review & editing. **Denis-Alexander Engemann:** Writing – review & editing, Methodology. **Amandine Pedoux:** Investigation. **Anna Bánki:** Writing – review & editing. **Florence Baillin:** Investigation. **Benjamin Landman:** Investigation. **Anna Maruani:** Investigation, Data curation. **Frederique Amsellem:** Investigation. **Thomas Bourgeron:** Funding acquisition, Project administration. **Richard Delorme:** Investigation, Project administration, Writing – review & editing. **Guillaume Dumas:** Conceptualization, Methodology, Supervision, Writing – review & editing, Visualization.

## Declaration of Competing Interest

The authors declare the following financial interests/personal relationships which may be considered as potential competing interests: D.E. is a full-time employee of F. Hoffmann-La Roche Ltd. All other authors report no biomedical financial interests or potential conflicts of interest.

## Data Availability

The data are not publicly available due to ethical restrictions. The codes used for the analyses are open source and available at https://github.com/ppsp-team/SoNeTAA/tree/master/HDC/EEG.
