# Supplementary material for: Cortico-Cerebellar neurodynamics during social interaction in Autism Spectrum Disorders
Source: Neuroimage Clin. 2023 Jun 28;39:103465. doi: 10.1016/j.nicl.2023.103465 (PMC10368923; doi:10.1016/j.nicl.2023.103465)
Supplement: Supplementary data 1 [file mmc1.docx]

# Sup. Figure 1


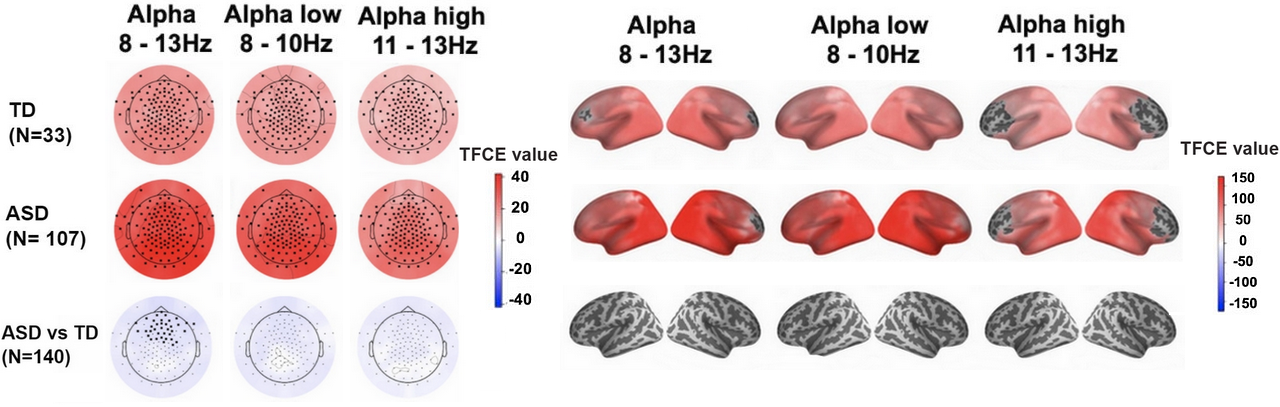


**Sup. Figure 1.** Scalp (left panel) and cerebral cortex (right panel) statistical maps during RS ‘eyes-closed’ compared to the RS ‘eyes-open’ condition for the alpha band (8-13 Hz), low alpha band (8-10 Hz), and high alpha band (10-13 Hz). In both panels, the upper, middle, and lower rows show TFCE values for the TD and ASD groups, and the ASD *vs* TD groups comparison (respectively), thresholded at p < 0.05, TFCE corrected. In the upper and middle rows, positive TFCE values indicate alpha enhancement during RS ‘eyes-closed’ vs RS ‘eyes-open’ in the TD and ASD groups (respectively). In the lower row, negative TFCE values indicate lower alpha enhancement during RS ‘eyes-closed’ in the ASD *vs* TD groups.

**Sup. Figure 2**


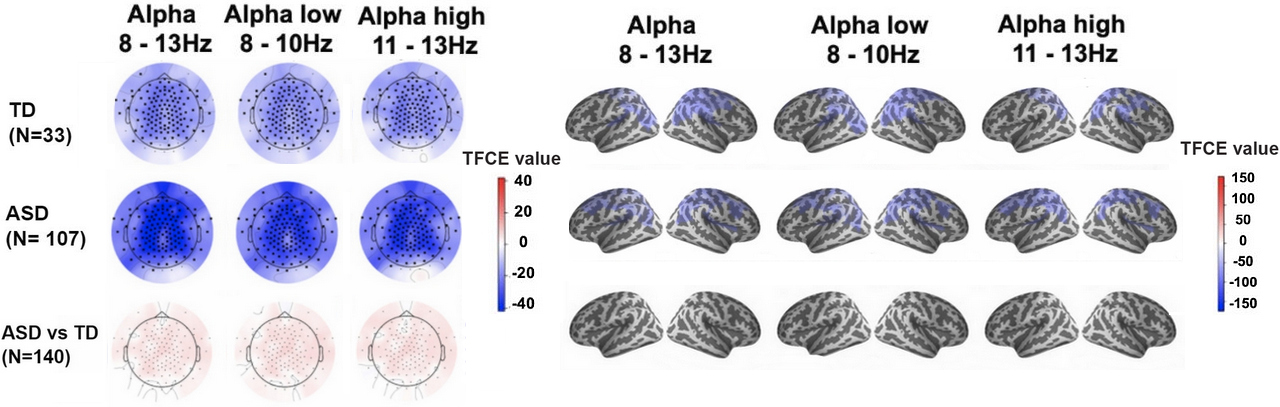


**Sup. Figure 2.** Scalp (left panel) and cerebral cortex (right panel) statistical maps during the HDC compared to the RS ‘eyes-open’ condition for the alpha (8-13 Hz), low alpha (8-10 Hz), and high alpha (11-13 Hz) bands. In both panels, the upper, middle, and lower rows show TFCE values for the TD and ASD groups, and the ASD *vs* TD groups comparison (respectively), thresholded at p < 0.05, TFCE corrected. In the upper and middle rows, negative TFCE values indicate alpha suppression during HDC *vs* RS in the TD and ASD groups (respectively). In the lower row, positive TFCE values indicate greater alpha enhancement during the HDC task in the ASD *vs* TD groups.

**Sup. Figure 3**


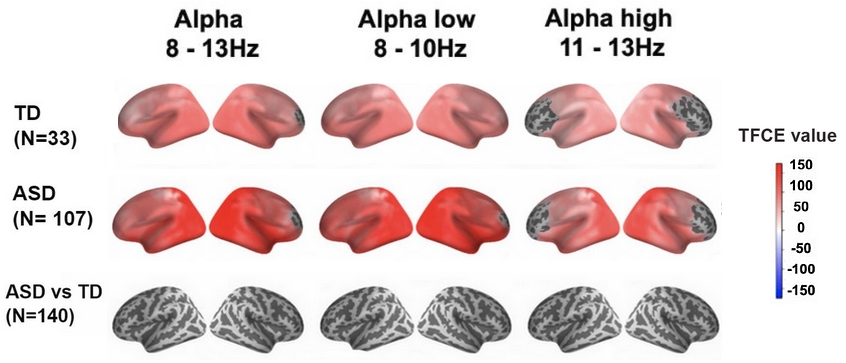


**Sup. Figure 3.** Cerebral cortex statistical maps during RS ‘eyes-closed’ compared to the RS ‘eyes-open’ condition for the alpha band (8-13 Hz), low alpha band (8-10 Hz), and high alpha band (11-13 Hz) after source reconstruction with the MNE algorithm. Statistical maps are similar to that obtained after source reconstruction with the eLORETA algorithm (Sup. Figure 1, right panel). The upper, middle, and lower rows show TFCE values for the TD and ASD groups, and the ASD *vs* TD group comparison (respectively), thresholded at p < 0.05, TFCE corrected. In the upper and middle rows, positive TFCE values indicate alpha enhancement during RS ‘eyes-closed’ vs RS ‘eyes-open’ in the TD and ASD groups (respectively). In the lower row, negative TFCE values indicate lower alpha enhancement during RS ‘eyes-closed’ in the ASD *vs* TD groups.

**Sup. Figure 4**


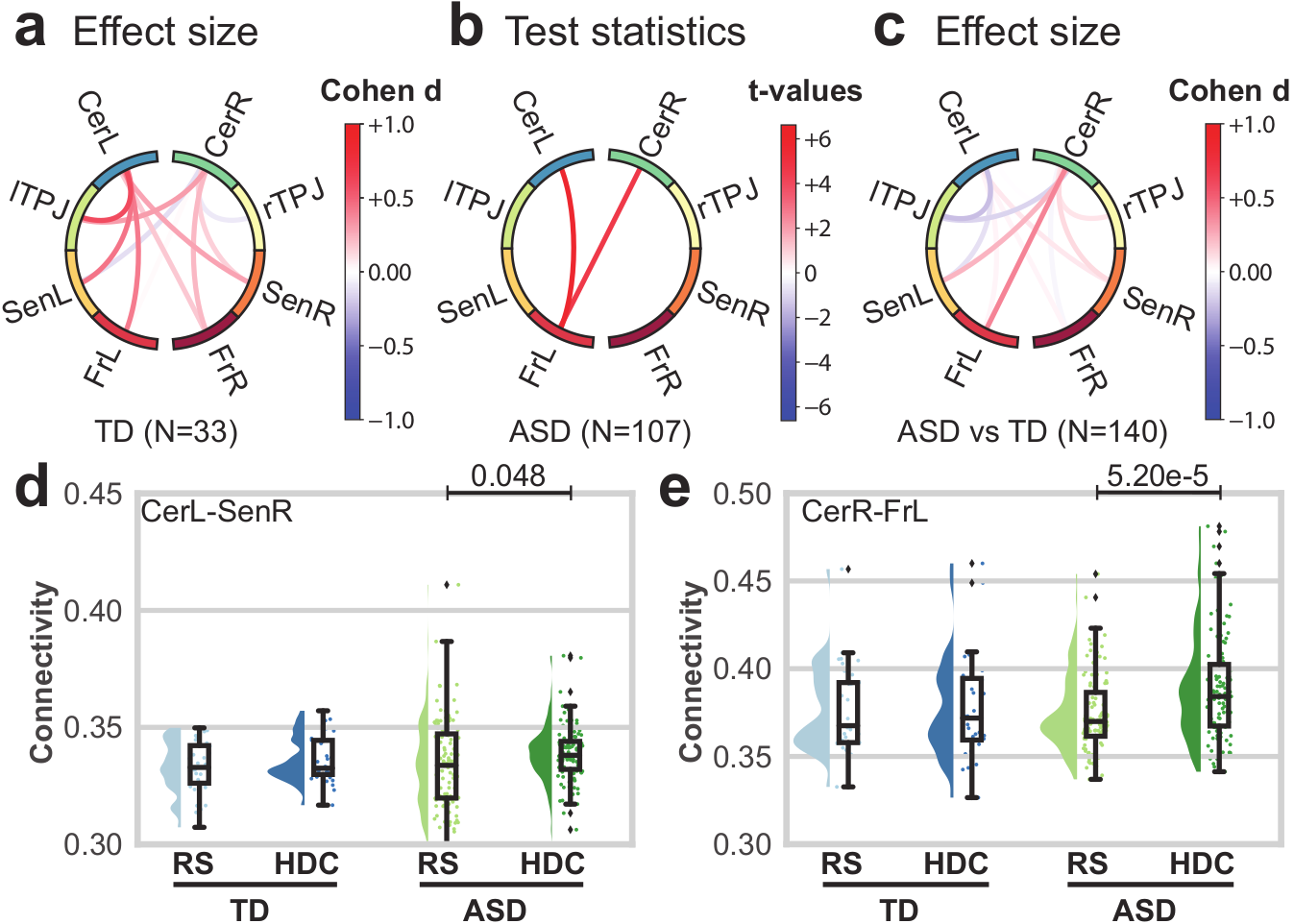


**Sup. Figure 4.** We replicated the coherence analysis after correction for spatial leakage. During the HDC task, the connectivity effect sizes and statistical plots were compared to those obtained in the RS ‘eyes-open’ condition for the theta band (3-8 Hz). Panels (a), (b), and (c) display a circle plot with connections between different brain areas, from top to bottom: cerebellar cortices (CerL/CerR), temporo-parietal junctions (lTPJ/rTPJ), sensorimotor cortices (SenL/SenR), and frontal cortices (FrL/FrR). Panels (a) and (c) show effect sizes for the TD and ASD *vs* TD groups (respectively). Panel (b) shows connectivity T-values for the group with ASD thresholded at p < 0.05, FDR corrected. On the panel (d), half violin plots display connectivity values between the left cerebellar cortex and the right sensorimotor cortex in the TD (blue) and ASD (green) groups during RS (light) and HDC (dark). On panel (e), half violin plots display connectivity values between the right cerebellar cortex and the left frontal cortex rTPJ in the TD (blue) and ASD (green) groups during RS (light) and HDC (dark). For each group, a density plot and a boxplot are displayed on the left and right (respectively). P-values indicate the results of intra-group comparisons (thresholded at p < 0.05, non-corrected).

**Sup. Figure 5**


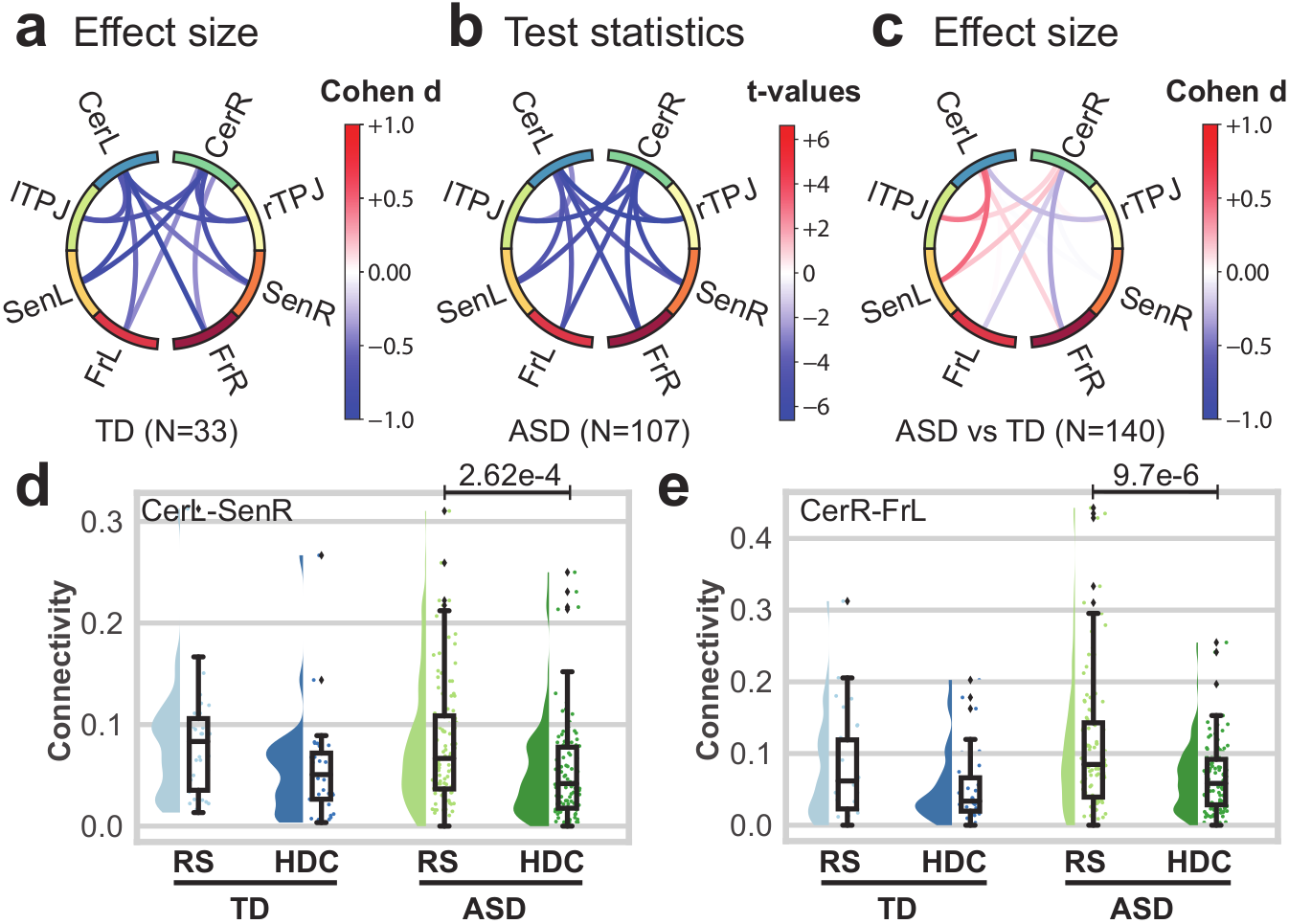


**Sup. Figure 5.** During the HDC task, the wPLI-based connectivity effect sizes and statistical plots were compared to those obtained in the RS ‘eyes-open’ condition for the theta band (3-8 Hz). Panels (a), (b) and (c) display a circle plot with connections between different brain areas, from top to bottom: cerebellar cortices (CerL/CerR), temporo-parietal junctions (lTPJ/rTPJ), sensorimotor cortices (SenL/SenR), and frontal cortices (FrL/FrR). Panels (a) and (c) show effect sizes for the TD and ASD *vs* TD groups (respectively). Panel (b) shows connectivity T-values for the group with ASD thresholded at p < 0.05, FDR corrected. On the panel (d), half violin plots display connectivity values between the left cerebellar cortex and the right sensorimotor cortex in the TD (blue) and ASD (green) groups during RS (light) and HDC (dark). On the panel (e), half violin plots display connectivity values between the right cerebellar cortex and the left frontal cortex rTPJ in the TD (blue) and ASD (green) groups during RS (light) and HDC (dark). For each group, a density plot and a boxplot are displayed on the left and right (respectively). P-values indicate the results of intra-group comparisons (thresholded at p < 0.05, non-corrected).
